# Processing of Spatial-Frequency Altered Faces in Schizophrenia: Effects of Illness Phase and Duration

**DOI:** 10.1371/journal.pone.0114642

**Published:** 2014-12-08

**Authors:** Steven M. Silverstein, Brian P. Keane, Thomas V. Papathomas, Kira L. Lathrop, Hristian Kourtev, Keith Feigenson, Matthew W. Roché, Yushi Wang, Deepthi Mikkilineni, Danielle Paterno

**Affiliations:** 1 Rutgers University Behavioral Health Care, Piscataway, New Jersey, United States of America; 2 Rutgers - Robert Wood Johnson Medical School, Department of Psychiatry, Piscataway, New Jersey, United States of America; 3 Rutgers University Center for Cognitive Science, New Brunswick, New Jersey, United States of America; 4 University of Pittsburgh School of Medicine, Department of Ophthalmology and Swanson School of Engineering, Department of Bioengineering, Pittsburgh, Pennsylvania, United States of America; 5 Albright College, Psychology Department, Reading, Pennsylvania, United States of America; University of Copenhagen, Denmark

## Abstract

Low spatial frequency (SF) processing has been shown to be impaired in people with schizophrenia, but it is not clear how this varies with clinical state or illness chronicity. We compared schizophrenia patients (SCZ, n = 34), first episode psychosis patients (FEP, n = 22), and healthy controls (CON, n = 35) on a gender/facial discrimination task. Images were either unaltered (broadband spatial frequency, BSF), or had high or low SF information removed (LSF and HSF conditions, respectively). The task was performed at hospital admission and discharge for patients, and at corresponding time points for controls. Groups were matched on visual acuity. At admission, compared to their BSF performance, each group was significantly worse with low SF stimuli, and most impaired with high SF stimuli. The level of impairment at each SF did not depend on group. At discharge, the SCZ group performed more poorly in the LSF condition than the other groups, and showed the greatest degree of performance decline collapsed over HSF and LSF conditions, although the latter finding was not significant when controlling for visual acuity. Performance did not change significantly over time for any group. HSF processing was strongly related to visual acuity at both time points for all groups. We conclude the following: 1) SF processing abilities in schizophrenia are relatively stable across clinical state; 2) face processing abnormalities in SCZ are not secondary to problems processing specific SFs, but are due to other known difficulties constructing visual representations from degraded information; and 3) the relationship between HSF processing and visual acuity, along with known SCZ- and medication-related acuity reductions, and the elimination of a SCZ-related impairment after controlling for visual acuity in this study, all raise the possibility that some prior findings of impaired perception in SCZ may be secondary to acuity reductions.

## Introduction

The presence of visual processing impairments is now well established in schizophrenia [1]–[5], and evidence is increasing for their etiologic [6], [7] and functional [8], [9] significance. A relatively unaddressed question in this literature, however, is whether (or which of) these impairments are state-linked, reflecting processes involved in the acute phase of illness, versus having trait status, possibly reflecting the diathesis for the disorder. Emerging evidence suggests that visual processing that is strongly driven by top-down modulation involving prior experience is state-linked [10]–[13] whereas processing that reflects primarily bottom-up influences is stable across clinical state [14]. However, there is little to no data on this issue for most visual processes that have been studied in schizophrenia.

One well-studied visual function in schizophrenia is spatial frequency processing. Much evidence now suggests that schizophrenia patients show a differential impairment in processing low spatial frequencies (LSFs) [15], [16], but this is not a universal finding. For example, some studies suggest a bias *towards* LSF processing when viewing faces, suggesting a problem integrating information across spatial frequencies [17], [18]. This conclusion is consistent with research on faulty integration of magnocellular and parvocellular pathway information in schizophrenia [19]. Others studies suggest that LSF information is detected normally but processed excessively, leading to disruptions in higher level visual tasks [20]. Still others indicate that the processing of all spatial frequencies (SFs) is impaired in schizophrenia and that this reflects attentional dysfunction rather than a primary visual impairment [21]. Some studies indicating reduced LSF processing have also found increased high spatial frequency (HSF) processing [22]–[24], while still others have demonstrated a specific impairment in processing HSFs [25]. It is not clear what the explanation is for this range of findings, although it is likely to reflect several factors. One is differences in the tasks used. A second is differences between studies in whether stimuli were presented at threshold or were suprathreshold. In general, LSF processing, and its corresponding putative neurobiological basis in magnocellular pathway activity, is most accurately measured at low contrast, threshold levels [26], [27]. A third factor is differences in visual acuity between groups. Schizophrenia patients are known to have poorer acuity than the general population [28], [29], and this would be expected to reduce fine detail (i.e., HSF) processing and create a bias towards LSF processing. Alternatively, it could create the appearance of difficulties at all spatial frequencies whereas, if acuity were optimized via an adequate prescription for corrective lenses, only an LSF impairment would remain (presumably reflecting the true nature of visual system pathophysiology). A fourth factor is the clinical characteristics of patients studied - schizophrenia is known to be a heterogeneous condition and factors such as level of premorbid functioning, and degrees of positive, negative, and disorganized symptoms have each been shown to be associated with performance on different perceptual tasks in patient samples in past studies [2], [10], [30]–[34]. In addition, first episode psychosis has been characterized by heightened LSF processing [23], [35], [36], which may be an aspect of a general hyperactivity in brain networks [37] that is no longer present in more chronically ill patients, who have often shown reduced LSF processing [38], [39].

Furthermore, as noted above, it is unclear whether these SF impairments are state-related. O'Donnell found impaired discrimination of sinusoidal luminance gratings at low, but not high, SFs in medicated schizophrenia patients [40], and Kiss et al. [14] replicated this finding in a sample of remitted, unmedicated, high functioning outpatients with schizophrenia who also demonstrated normal IQs and intact attentional functioning. These studies suggest that the LSF processing impairment is a trait-like visual processing deficit in schizophrenia. To our knowledge, however, there has been only a single study examining SF processing longitudinally in the same patients as they move from the acute to the stabilization phase of illness (following the clinical state distinctions of Lehman et al [41]). Kelemen et al. [23] studied medication naïve first episode schizophrenia patients at baseline (whether or not the person was in the hospital at baseline testing was not reported) and after 8 weeks of treatment with an antipsychotic medication. Excessive LSF processing was noted at baseline, but not at follow-up, suggesting that medication normalizes hyperactive LSF processing and magnocellular pathway activity, at least in first episode patients. In contrast to Kelemen et al., in the present study, all first episode patients were hospitalized and on medication at the time of the initial assessment, and later-episode patients were included in the study.

Specifically, we examined the effects of SF manipulations on emotionally neutral face processing in the context of performing a gender-discrimination task that had been used in a prior fMRI study of SF removal effects on face processing in schizophrenia [24]. We chose a gender-discrimination task for several reasons, including: (1) gender discrimination is a relatively simple task and is therefore unlikely to introduce confounds from a generalized deficit, reduced motivation and/or anxiety due to failure experiences [42]; (2) ERP studies in humans and monkeys demonstrate that gender discrimination tends to occur separately from and faster than the structural encoding responsible for detection of identity or expression [43]–[45]; and (3) gender processing does not seem to interfere with detection of either facial features or global processing of faces [45]. We manipulated the SF composition of faces in two ways. First, to increase the salience of global form (LSF condition), we removed HSF. Second, to increase the salience of local contour information (HSF condition), we removed LSF. The rationale for these manipulations is supported by studies indicating that when forced to rely on either high or low SF data alone for face identification, healthy adults and children above 8 months of age tend to rely on low SF information, which may represent a tendency to initially encode the global structure of the face to determine identity [42], [46] [47], [48]. Recent ERP data also confirm the association between global and LSF processing, and local and HSF processing [49].

A second purpose of the study was to compare people with an established diagnosis of schizophrenia to people experiencing a first episode of psychosis (many of whom will likely be diagnosed later as having schizophrenia [50], [51]) to determine the degree to which task performance is affected by illness progression. This is an important question because while some aspects of perceptual impairment can be detected in first-episode patients [35], [52], [53], others emerge over time [12], [54], [55] and may thus reflect progressive changes in brain structure or function (e.g., related to loss of gray and white matter in the occipital cortex [56]). There is also, as noted above, evidence suggesting that extent of LSF processing may shift from being excessive to being reduced from first to later illness episodes, although, to our knowledge, there are no published data directly comparing first- and later-episode patients on the same task of SF processing. In addition, whether first- and later-episode patients would demonstrate similar degrees of change in performance during recovery from an acute psychotic episode has not been reported.

## Method

### Subjects

One hundred twenty subjects (FEP = 29; SCZ = 48; CON = 43) completed data collection at the initial (admission) testing (i.e., Time 1). Of these, ninety-four subjects completed the gender discrimination task at both time points (23 FEP, 36 SCZ, 35 CON). Attrition was due to lack of interest in completing the second session, and, for some patients, sudden hospital discharge or transfer to another hospital. In addition, data from 1 FEP and 2 SCZ subjects were excluded for performing below chance (see below, Data analysis strategy), at one or both time points, in the BSF (easiest) condition, leaving final sample sizes of 22 FEP, 34 SCZ, and 35 CON (see Table 1 for gender and demographic distributions within groups). Patients were tested as close as possible to their hospital admission and discharge dates. Controls were tested at corresponding time points. All patient diagnoses were confirmed with the Structured Clinical Interview for DSM-IV Diagnosis (SCID), patient version [57], and symptoms were assessed with the Positive and Negative Syndrome Scale [58], which was scored using a 5-factor model (positive, negative, cognitive, excitement, depression) [59], [60]. Additionally, a separate factor was derived for disorganized symptoms [61] that consisted of the original PANSS items conceptual disorganization and poor attention, and an added item for inappropriate affect [61]. The absence of diagnosable clinical conditions in the CON group was established using the SCID, non-patient version [62]. Socioeconomic status (SES) was determined for all subjects, and their parents (via subject report), using an updated version of the Nakao-Treas scale [63].

**Table 1:** Demographic data.

	FEP (n = 22)	SCZ (n = 34)	CON (n = 35)
Age	26.32(9.82)	40.09(10.90)	43.74(11.97)
Gender	11 M/11 F	22 M/12 F	16 M/19 F
Race	10 W/5 AA/7 A	15 W/13 AA/6 A	15 W/13 AA/7 A
Ethnicity	19 NH/3 H	28 NH/5H	30 NH/5 H
Education	13.09(2.89)	13.06(1.65)	14.06(2.31)
SES	47.02(20.80)	36.36(14.62)	53.78(17.97)
Maternal Education	13.33(4.29)	12.65(4.42)	12.49(4.56)
Paternal Education	13.80(3.82)	13.04(4.20)	12.09(4.84)
Maternal SES	53.63(22.28)	47.18(20.95)	53.15(21.03)
Paternal SES	57.03(21.18)	52.48(20.91)	52.02(24.29)
Acuity – Left eye	.08(.10)	.13(.13)	.11(.11)
Acuity – Right eye	.12(.15)	.14(.13)	.13(.13)
Acuity – Both eyes	.06(.08)	.11(.14)	.09(.12)

To be included in the study, patients had to be between the ages of 18–60, and diagnosed with either schizophrenia, or a first episode of a psychiatric disorder with psychotic symptoms. Exclusion criteria included: (1) any history of TBI or head injury with loss of consciousness greater than 10 min; (2) history of a neurological or developmental disorder; (3) current mood disorder; (4) current substance abuse or dependence disorder (within past 6 months) or positive urine toxicology screen on any day of testing; (5) estimated premorbid (Wechsler) IQ<70, as determined by the Shipley Institute of Living Scale [64] or evidence of intellectual disability as indicated in the electronic medical record; or (6) ECT within the past 8 weeks. All patients were receiving antipsychotic medication. Exclusion criteria for the CON group included those listed for patients, as well as: (1) any lifetime DSM-IV Axis-I disorder (as assessed by SCID) with the exception of past substance use disorders; (2) psychotropic medication use in the last 6 months; and (3) first-degree relative(s) with a diagnosis of schizophrenia, schizoaffective disorder, or bipolar disorder (based on subject self report). All subjects had normal or corrected-to-normal visual acuity as assessed via a Snellen chart. For subjects who initially had poor acuity (poorer than 20/40 Snellen values), corrective lenses (Vision Correction Lenses, Psychology Software Tools, Inc., Pittsburgh, PA, 15215) were worn to optimize acuity to the extent possible.

The study was approved by the IRB at Rutgers-Robert Wood Johnson Medical Center. All investigators completed on-line training in responsible and ethical conduct of research at their respective institutions. All subjects provided written informed consent, and were deemed to have the capacity to provide consent. The latter was ensured by: 1) requiring referrals to the study to be made by inpatient staff members, and to be limited only to patients they believed had the capacity to understand the study and the voluntary nature of participation; 2) having trained research assistants review all sections of the consent form with the patient prior to the latter signing it; and 3) after the review of the consent form, asking the patient a series of three IRB-approved questions (listed at the end of the consent form) to ensure that they understood the purpose of the study, the tasks they would be completing, and the voluntary nature of participation, including the lack of any penalties for withdrawal at any time. Patient responses were compared to IRB-approved response alternatives that were considered to meet criteria for comprehension of the study and its procedures. Only patients who completed all three questions correctly and who then provided written informed consent were allowed to participate. Patient responses were recorded on the consent form and are available for IRB audit.

### Stimuli and procedure

**Figure 1:**
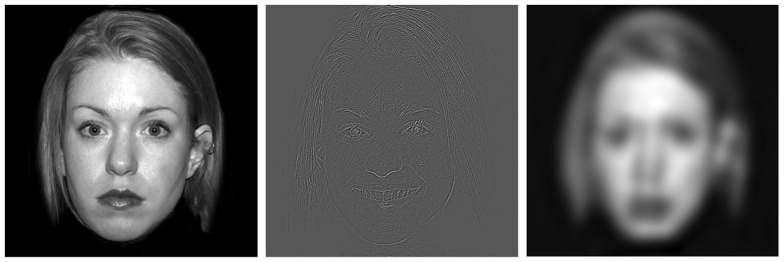
Examples of BSF (left), HSF (center), & LSF (right) stimuli from gender discrimination task.

Stimuli were black-and-white photographs of emotion-neutral faces taken from the Nim Stim facial database [65]. Background information was removed although the hair remained. There was a good distribution of different genders and ethnicities although there were not enough images to completely balance race and gender for all conditions. Represented groups include African-American Males, White Males, Asian Males, African-American Females, White Females and Asian Females. The stimulus conditions were: 1) normal or broad spatial frequency (BSF) faces (i.e., unmanipulated images); 2) HSF faces, which contained images with low frequency signals removed; and 3) LSF faces, which contained images with high frequency signals removed. The spatial-frequency content in the original images was altered using procedures similar to those of Bar and colleagues for fMRI studies of HSF and LSF processing [66]. Specifically, using the Image Processing Toolkit and PhotoshopCS (Adobe, USA), images were transformed into Fourier space and thresholded with a bilevel threshold tool. LSF images were thresholded to 1.790 cycles/degree and HSF images were thresholded to 7.517 cycles/degree. Images were then smoothed using a 5-pixel-radius Gaussian filter and were converted back into image space. An example stimulus from each condition is shown in Fig. 1.

Across 108 trials [36 in each condition: BSF, HSF, LSF], after presentation of a facial image, subjects had to press one key if they perceived the figure to be a male, and another key if they perceived the figure to be female. Face stimuli appeared centrally on the screen for 325 milliseconds, and the trial continued until the subject responded (to ensure a response on each trial). Following a participant's response, there was a 1-second interval before the next trial began. The 108 stimuli were broken up into 2 blocks of 54 stimuli. Stimuli from each condition were pseudo-randomly intermixed (in equal numbers) in each block, with randomization varying across subjects. There was a 30-second break between blocks. Prior to data collection, there were 12 practice trials, which were composed of 4 stimuli from each condition, to ensure that subjects understood the task. The entire face perception task took approximately 6 minutes to complete.

Stimuli were presented on a Samsung 2243BWX LCD monitor with viewable dimensions of 47.5 by 29.8 cm. The viewing distance was 24 inches (60.9 cm), and therefore, the viewable screen subtended 37.95°×26.07° of visual angle. The screen resolution was 1680×1050 pixels. Each image was 10.5 cm high ×7 cm wide, thus subtending 9.78° by 5.56° of visual angle. Images presented in the experimental task were scaled to 82% of their original size, and so the spatial frequency of the LSF images was 2.17 cycles/degree, and of the HSF images was 9.10 cycles/degree.

Spyder 3 Elite software was used to calibrate the monitors at the start of the study and then monthly afterwards. Monitor parameters were a gamma value of 2.2, color temperature (white point) of 6500K, and luminance of 120 cd/m^2^.

### Data analysis strategy

Data were included for subjects who scored at least 61.11% correct (or 22 out of 36 correct) in the BSF condition, at both time points. Under a binomial distribution, with 36 trials, 23 or more trials would be correct by chance less than 5% of the time. As noted above, this led to exclusion of data from 1 FEP and 2 SCZ subjects. Note, however, that the findings using the entire sample were essentially identical to what was observed with the 3 excluded subjects (see S1 Results). In S1 Results, we also present analyses using: 1) all subjects who scored above chance at Time 1 (i.e., admission) regardless of whether they were present at Time 2 (i.e., discharge), or (if present) their performance level at Time 2; 2) only subjects who scored above 90% accuracy in the BSF condition at Time 1; this ensured that the data reflected only subjects who could perform the task with a high degree of accuracy, thereby reducing confounds related to keypress errors and attention lapses [FEP mean = 96.50(SD = .03), SCZ = 96.11(.03) = , CON = 97.81(.03)]; and 3) patients who were present versus those who were not present at Time 2, on Time 1 variables to address the issue of drop-out bias.

To analyze the data on visual acuity, Snellen fractions (e.g., 20/40) were converted to logarithm of the minimum angle of resolution (LogMAR) units. The latter provide more accurate estimates of vision when using parametric statistics [67].

Antipsychotic medication dosages were converted to chlorpromazine (CPZ) equivalent units, following established procedures [68]. These values were then correlated with task performance indices to determine if cross-sectional relationships existed, and if medication change over time was related to any performance change over time. A second set of similar analyses were done using benzodiazepine dosages, which were also converted to standardized units, representing diazepam dosage [69].

Initial analyses compared the three groups on the BSF condition, as poorer performance in the patient groups (compared to the CON group) in this condition could be indicative of a generalized performance impairment. Since this difference was significant (see below), to remove its impact from our analyses, the critical indices for this study were calculated such that they represented the contrasts in performance between BSF and the LSF, and HSF, conditions (i.e., the relative performance *decrement* due to spatial frequency manipulation, in each of the 2 degraded conditions). Groups were compared on these 2 variables at each time point, and these indices were correlated with symptom factor scores in the patient groups to determine if performance was related to symptoms. We also calculated a differential sensitivity index which represented [(BSF-LSF) – (BSF-HSF)], to identify the relative effects of removing one SF type over the other. We then compared this between groups. On this differential sensitivity index, a more negative score would indicate a greater performance decrement from BSF in the HSF condition compared to the decrement from BSF in the LSF condition. A score of 0 would indicate equal performance decrement from BSF in both degraded conditions, and a positive score would indicate greater performance decrement from BSF in the LSF condition compared to the decrement from BSF in the HSF condition. Finally, we calculated an overall degradation index (i.e., decline in performance due to *any* type of stimulus degradation) by summing the degree of performance decrement in the LSF and HSF conditions, and then comparing groups on this variable. Data on both accuracy and reaction time (RT) (for correct responses) are reported.

## Results

### Demographic and clinical data (see Table 1)

#### Demographic data

The groups differed significantly in age: *F*(2, 90) = 17.50, *p*<.001. Post-hoc Scheffé tests indicated that, as expected, the FEP group was younger than both the SCZ and CON groups (*p*s <.001), who did not differ from each other (*p* = .40). The groups did not differ in terms of education attainment (*F*(2,90) = 2.06, *p* = .13), or composition in terms of gender [X^2^(2) = 2.67, *p* = .26], race [X^2^(4) = 2.44, *p* = .66], or ethnicity (Hispanic vs. non-Hispanic) [X^2^(2) = .03, *p* = .99]. There was a significant group difference on SES [*F*(2,56) = 6.55, *p* = .003], with the SCZ group having lower attainment than the CON group (*p* = .003), but not the FEP group (*p* = .29). The FEP and CON groups did not differ on SES (*p* = .63). There were no group differences in terms of maternal [*F*(2,86) = 0.25, *p* = .78] or paternal [*F*(2,81) = 1.00, *p = *.37] education, or maternal [*F*(2,60) = 0.65, *p* = .53] or paternal [*F*(2,66) = 0.33, *p*< = .72] SES status.

#### Time between testing sessions

The average duration between testing time points was 15.41 days (SD = 6.43; range = 6–35). There was a significant between-groups difference in the number of days between testing sessions: *F*(2,90) = 4.92, *p* = .009. Post-hoc Scheffé tests indicated that the number of days between testings for the SCZ group (mean = 12.82, SD = 5.01) was significantly less than for the CON group (17.31, 5.54) (*p* = 01). The SCZ and FEP (16.36, 8.38) groups did not differ (*p* = .12), nor did the FEP and CON groups (*p* = .85). Because the homogeneity of variance assumption was violated for the ANOVA, the groups were also compared using Kruskal-Wallis and median tests, which were also significant (*p*s = .001 and.03, respectively).

#### Visual acuity

The groups did not differ in acuity: left eye *F*(2,83) = 0.96, *p* = .34; right eye *F*(2,83) = 0.06, *p* = .94), binocular (*F*(2,83) = 0.94, *p* = .39).

#### Symptoms

At admission, the FEP and SCZ groups did not differ in their level of symptoms: positive *t*(52) = −.33, *p* = .74; negative *t*(52) = −1.25, *p* = .21; cognitive *t*(52) = −.82, *p* = .42; excitement *t*(52) = .81, *p* = .42; depression *t*(52) = −.95, *p* = .35; disorganization *t*(52) = −1.03, *p* = .31. At discharge, the groups were also similar: positive *t*(52) = −1.00, *p* = .32; negative *t*(52) = −.89, *p* = ..38; cognitive *t*(52) = −1.03, *p* = .31; excitement *t*(52) = −.12, *p* = .91; depression *t*(52) = −.40, *p* = .69; disorganization *t*(50.96) = −1.63, *p* = .11.

Analyses of change over time from admission to discharge, for both patient groups combined, indicated that symptoms were significantly reduced at Time 2 compared with Time 1, confirming clinical improvement between time points: positive *t*(51) = 6.15, *p*<.001; negative *t*(51) = 3.57, *p* = .001; cognitive *t*(51) = 3.67, *p* = .001; excitement *t*(51) = 4.03, *p*<.001; depression *t*(51) = 4.75, *p*<.001; disorganization *t*(51) = 3.86, *p*<.001. When analyzed for each patient group separately, this effect was significant [using MANOVAs with the first 5 (orthogonal) symptom factors included (i.e., the disorganization factor excluded)] for both the FEP [*F*(1,18) = 27.15, p≤.001) and SCZ [*F*(1,32) = 24.69, *p*<.001] groups. In neither case was there a significant time x symptom interaction: FEP [*F*(4,72) = 1.13, *p* = .35]; SCZ [*F*(4,128) = 1.00, *p* = .41]. The patient groups also did not differ on their degree of differential symptom change (across the 5 PANSS factors) over time: time x group x symptom interaction *F*(4,200) = .68, *p* = .61. There was also not a significant difference in degree of change over time on the Cuesta and Peralta disorganization factor: *F*(1,50) = .01, *p* = .92. All of this suggests that any group differences in task performance, either at a given time point, or across time, are not due to patient differences in symptomatology or treatment response.

### Task performance: BSF condition

**Figure 2:**
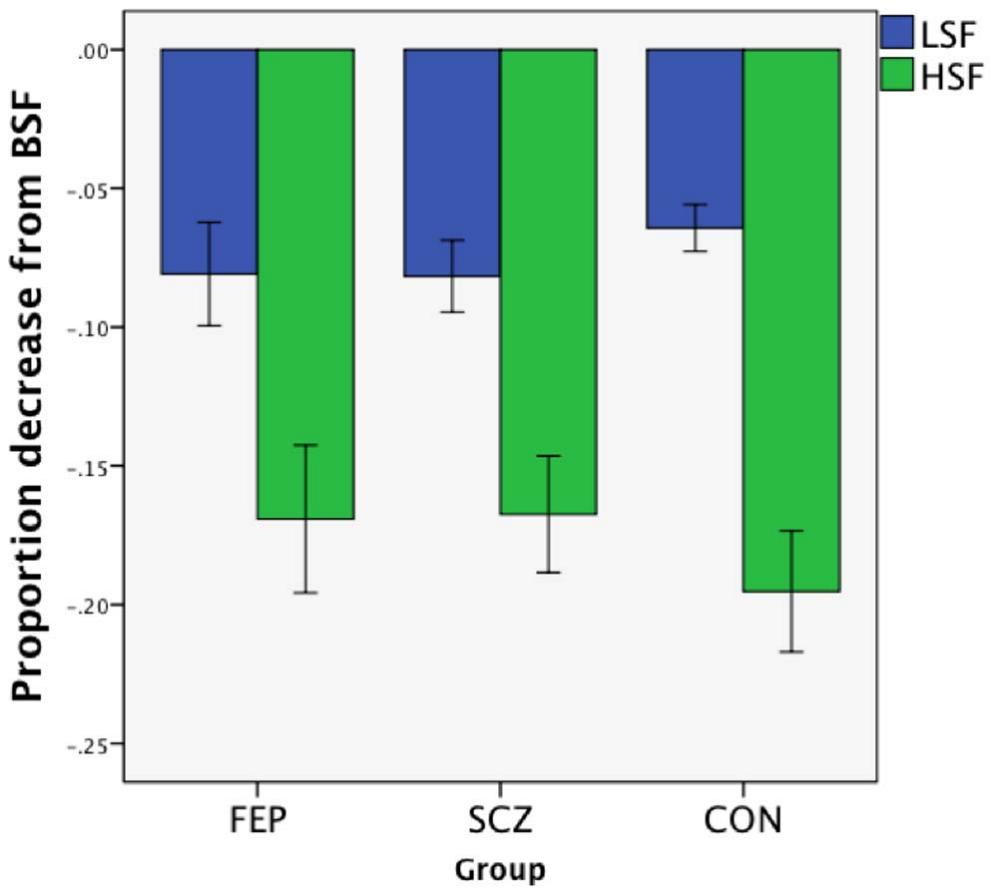
Performance decrement from BSF, in LSF and HSF conditions, for each group, at Time 1. Error bars reflect +/− 1 SE.

**Figure 3:**
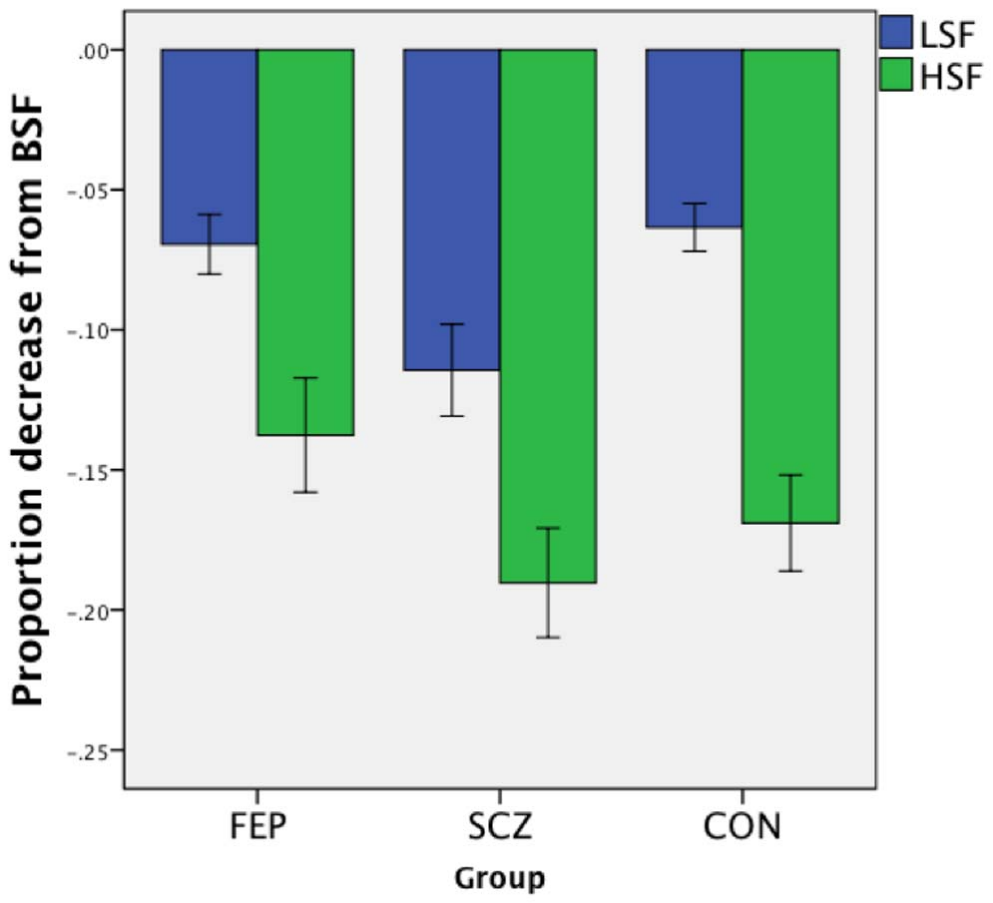
Performance decrement from BSF, in LSF and HSF conditions, for each group, at Time 2. Error bars reflect +/− 1 SE.

First, BSF performance was considered. Accuracy was high for all groups at each time point, considered separately. Time 1: FEP mean = .93 (SD = .08), SCZ = .90 (SD = .10), CON = .97 (SD = .04); Time 2: FEP = .92 (SD = .08), SCZ = .92 (SD = .07), CON = .97 (.03). This suggests that all groups were actively engaged in the task. However, there were main effects of group at both points: Time 1 *F*(2,90) = 6.75, *p* = .002; Time 2 *F*(2,90) = 6.23, *p* = .003. At Time 1, post-hoc Scheffé tests indicated that the SCZ group was less accurate than the CON group (*p* = .002), while no other pairwise comparisons were significant (both *p*s = .27). At Time 2, the FEP group performed more poorly than the CON group (*p* = .03), and the SCZ group performed more poorly than the CON group (*p* = .007), but the two patient groups did not differ from each other (*p* = .98). As noted above, because of these group differences in BSF performance, the primary data analyses focused on group-wise differences in the degree of performance decrement from the BSF condition, in the LSF and HSF conditions (see Figs. 2 and 3).

#### Performance at Times 1 and 2 in HSF and LSF conditions, relative to BSF

**Figure 4:**
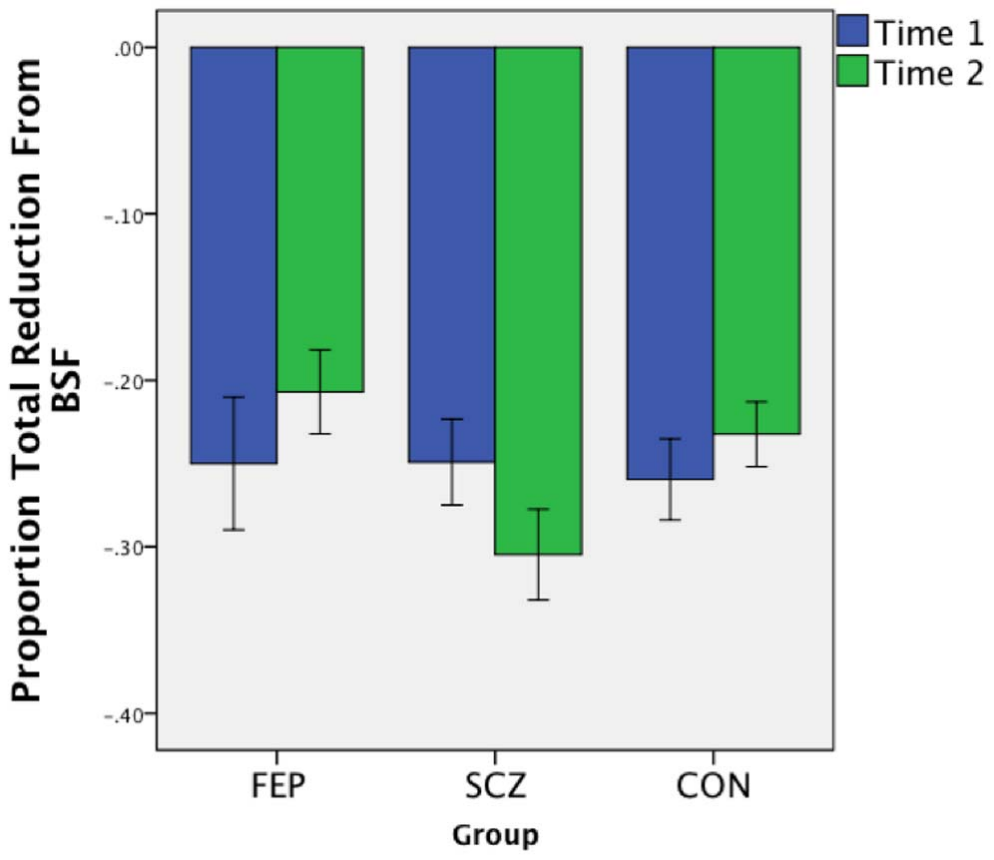
Overall degradation in performance from BSF (summed across HSF and LSF conditions), by group and time. Error bars reflect +/− 1 SE.

At Time 1, accuracy data indicated that the groups were equivalent in their decrease in performance (relative to BSF) in both the LSF [*F*(2,90) = 0.64, *p* = .53] and HSF [*F*(2,90) = 0.50, *p* = .61] conditions. The groups were also similar on the differential sensitivity [*F*(2,90) = 1.27, *p* = .29] and degradation [*F*(2,90) = 0.20, *p* = .82]] indices (see Fig. 4). Findings were similar when all subjects present at Time 1 were included in the analysis (i.e., when there was no BSF cut-off), when the cutoff for inclusion was 61.11% correct (i.e., above chance) at Time 1 only, and when it was 90% correct in BSF at Time 1 only (see S1 Results). This suggests that the accuracy results are not artifacts of attrition by a unique subgroup of subjects.

Time 1 RT data indicated that the groups did not differ on their degree of RT increase from BSF to HSF [*F*(2,90) = 0.77, *p* = .46]. However, there was a significant group difference on degree of RT change from BSF to LSF [*F*(2,90) = 4.80, *p* = .01]. The FEP group demonstrated the largest relative increase in RT in the LSF condition, and this was significantly larger than that demonstrated by the CON group (*p* = .01). The FEP group did not differ from the SCZ group (*p* = .14), and the SCZ and CON groups did not differ on this variable (*p* = .49). On the sensitivity RT index, there was a significant effect of group: *F*(2,90) = 3.16, *p*<.05. The FEP group showed the least differential sensitivity between the LSF and HSF conditions, although no pairwise comparisons reached statistical significance: FEP <SCZ, *p*<.08; FEP <CON, *p* = .09; SCZ = CON, *p*>.99. The groups did not differ in their overall performance decline when SF information was removed: degradation RT index *F*(2,90) = 1.98, *p* = .14. Findings were similar when all subjects present at Time 1 were included in the analysis (i.e., when there was no BSF cut-off), when the cutoff for inclusion was 61.11% correct at Time 1 only, and when it was 90% correct in BSF at Time 1 only (see S1 Results). This suggests that the RT results are not artifacts of attrition by a unique subgroup of subjects.

At Time 2, there was again no group difference in HSF condition accuracy relative to BSF: *F*(2,90) = 1.69, *p* = .19. However, the groups differed in their degree of performance decline in the LSF condition: *F*(2,90) = 5.09, *p* = .008. Post-hoc Scheffé tests indicated that the largest degree of performance decline was in the SCZ group (mean = −11%(SD = 10%)). This was significantly different than the performance of the CON group (−6% (5%); *p* = .01), and approached a significant reduction compared to the FEP group (−7%(5%); *p* = .07). There was no group difference on the sensitivity index [*F*(2,90) = 0.84, *p* = .43], but the groups differed significantly on the degradation index [*F*(2,90) = 4.25, *p*<.02]. Post-hoc Scheffé tests indicated that at Time 2, the SCZ group demonstrated more overall performance impairment when SF information was removed from the facial images, compared to the FEP (p<.03) group, with a trend towards poorer performance than the CON (p<.09) group, while the FEP and CON groups did not differ from each other (*p* = .79). Findings were similar when the Time 2 data set was restricted to only those subjects scoring above 90% in the BSF condition at Time 2, with the notable exception that the SCZ group was no longer significantly worse in in the LSF condition (see S1 Results). These findings, with the larger data set, are further evidence that the results from the restricted data set are not influenced by subject attrition.

Time 2 RT data indicated that the groups did not differ on their degree of RT increase from BSF to HSF [*F*(2,90) = 0.69, *p* = .50]. The groups were also equivalent on their degree of RT change from BSF to LSF [*F*(2,90) = 1.72, *p* = .19], and on the sensitivity [*F*(2,90) = 0.62, *p* = .54], and degradation [*F*(2,90) = 0.88, *p* = .42] RT indices.

#### Change over time

In the accuracy data, examined across time points, there was a significant main effect of condition, with accuracy in the HSF condition (relative to BSF) declining more than that in the LSF condition (relative to BSF): *F*(1,88) = 66.73, *p*<.001. No other accuracy effects were significant: time *F*(1,88) = 0.07, *p* = .79; group F(2,88) = 1.22, p = .30; group x time *F*(2,88) = 2.95, *p*<.06; condition x group *F*(2,88) = 1.43, *p* = .25; time x condition *F*(1,88) = 1.77, *p* = .19; time x condition x group *F*(2,88) = 0.13, *p* = .88. Exploration of the trend toward a group x time interaction revealed non-significant effects of time for the FEP [*F*(1,21) = 0.99, *p* = .33] and CON [*F*(1,34) = 1.67, *p* = .21] groups, and a trend towards a significant effect for the SCZ group (with relative performance declines from BSF being larger at Time 2 than at Time 1): *F*(1,33) = 2.95; *p*<.10. On the differential sensitivity index, there were no significant effects: time *F*(1,88) = 1.77, *p* = .19; group *F*(2,88) = 1.43, *p* = .25; time x group interaction: F(2,88) = 0.13, p = .88. On the degradation index the effects of time [*F*(1,88) = 0.07, *p* = .79] and group [*F*(2,88) = 1.22, *p* = .30] were not significant. However, there was a trend towards a significant time x group interaction: *F*(2,88) = 2.95, *p*<.06. Exploration of this effect revealed non-significant effects of time for the FEP [*t*(21) = 1.00, *p* = .33] and CON [*t*(34) = 1.29, *p* = .21] groups, and a trend towards an effect of time for the SCZ group [*t*(33) = −1.72, *p*<.10].

In the RT data, examined across time points, there was a significant main effect of condition, with the relative increase in RT in the HSF condition exceeding that in the LSF condition: *F*(1,88) = 14.78, *p*<.001. No other RT effects were significant: time *F*(1,88) = 0.04, *p* = .84; group F(2,88) = 2.06, *p* = .13; time x group *F*(2,88) = 0.12, *p* = .89; condition x group *F*(2,88) = 1.25, *p* = .29; time x condition *F*(1,88) = 0.73, *p* = .40; time x condition x group *F*(2,88) = 0.65, *p* = .53. There were also no significant RT effects on the sensitivity index: time [*F*(1,88) = 0.73, *p* = .40; group *F*(2,88) = 1.25, *p* = .29; time x group *F*(2,88) = 0.65, *p* = .53], or the degradation index [time *F*(1,88) = 0.04, *p* = .84; group *F*(2,88) = 2.06, *p* = .13; time x group *F*(2,88) = 0.12, *p* = .89].

### Visual acuity and performance

Because poorer visual acuity, by definition, reduces the resolution of fine detail (e.g., HSF information), we evaluated the relationship between visual acuity and task performance. Data presented here are for binocular acuity, but the results are similar for left or right eye acuity only (see Table 1 for values). There were significant correlations between visual acuity and all accuracy indices involving the HSF condition, and in all cases, poorer acuity was associated with a greater performance decline in the HSF relative to the BSF or LSF condition: BSF-HSF decline at Time 1 *r*_s_ = −.36, *p*<.001; BSF-HSF decline at Time 2 *r*_s_ = −.43, *p*<.001; sensitivity at Time 1 *r*_s_ = −.29, *p* = .008; sensitivity at Time 2 *r*_s_ = −.35, *p* = .001; degradation at Time 1 *r*_s_ = −.28, *p* = .01; degradation at Time 2 *r*_s_ = −.36, *p* = .001. There were no relationships between visual acuity and performance in the LSF condition alone at Time 1 (*r*_s_ = .00, *p*>.99] or Time 2 (*r*_s_ = −.02, *p* = .89].

The relationships reported above were weaker for the RT data, where the only significant result was with LSF-related accuracy decline at Time 1, and this result was modest and would not survive correction for multiple correlations: BSF-HSF decline at Time 1 *r*_s_ = −.07, *p* = .51; BSF-HSF decline at Time 2 *r*_s_ = −.05, *p* = .067; sensitivity at Time 1 *r*_s_ = .19, *p* = .09; sensitivity at Time 2 *r*_s_ = .04, *p* = .72; degradation at Time 1 *r*_s_ = .02, *p* = .86; degradation at Time 2 *r*_s_ = −.08, *p* = .50; BSF-LSF decline at Time 1 *r*_s_ = .27, *p* = .01; BSF-LSF decline at Time 2 *r*_s_ = .02, *p* = .85]. The RT findings also indicate no evidence of speed-accuracy trade-off.

Because the groups did not differ in overall visual acuity, while acuity was related to performance within the sample as a whole, analyses of covariance – controlling for visual acuity - on the between-group tests were appropriate [70]. In all cases except one, the results were qualitatively the same. The sole exception was for the group difference on the degradation index at Time 2, which was no longer significant: *F*(2, 83) = 2.12, *p* = .13.

### Symptom change and correlates of performance

Few of the correlations between task performance and symptoms were significant, for the patient group as a whole or for each group separately. Moreover, no clear pattern emerged, and none of the significant values would survive correction for multiple tests. These analyses are reported in S1 Results.

### Medication and performance

Data on changes in medication dosages over time, for both antipsychotic medications and benzodiazepines, are presented in S1 Results. Data on correlations between medication dosages (CPZ and diazepam equivalent) and sensitivity and degradation (accuracy) index scores at Time 1 and Time 2, as well as correlations between change in medication dosages over time and changes on these task indices, are also presented in S1 Results. Out of all correlations performed (for both patient groups separately and for both patient groups combined), only one value was significant, and this would not survive correction for multiple comparisons. We conclude therefore that task performance was unrelated to medication dosage.

## Discussion

The primary question addressed by this study was whether high and low SF processing abilities, as reflected in face processing, change as schizophrenia patients move from the acute to the stabilization phase of illness. We did not find evidence of significant improvement over time in the SCZ group, although there was a trend indicating that these patients were *more* sensitive to changes in spatial frequency composition of facial stimuli as they began recovering from acute illness. Subject attrition cannot account for the Time 1 results (i.e., the results of our analyses restricting the data set to only subjects who were present at both time points). This is because the same results were obtained with larger and smaller versions of the data set, which both included and excluded subjects present at Time 2 in the Time 1 analyses, and used 2 different performance criteria cutoffs (61.11% and 90% in the BSF condition). Moreover, a direct comparison of subjects who were present versus not present at Time 2 revealed no differences on performance variables at Time 1. However, the possibility that some of our results at Time 2 could be due to loss of subjects who were only present at Time 1 cannot be completely ruled out. It is possible, for example, that the trend towards a relative worsening over time for the SCZ group is, in part, an artifact of attrition. As noted above, however, there was no evidence that subjects who left the study after the admission testing performed differently on any variable than did subjects who were tested at both time points. It is also possible that the appearance of greater impairment for the SCZ group at Time 2 is a statistical artifact. Specifically, on the 2 variables where the SCZ group performed most abnormally (LSF accuracy decline from BSF, and overall performance degradation after removal of SFs), the SCZ group demonstrated small increases in SD from Time 1 to 2, whereas the FEP group demonstrated ∼40% reductions in SD across time points, and the CON group stayed the same (LSF) or declined slightly (degradation). We also consider it unlikely that the observed pattern of results was due to the SCZ group having the shortest time between testing sessions of all the groups. This is because the two patient groups did not differ on symptoms at either time point, or on degree of symptom change across time points, and because the slight worsening of SCZ group performance over time occurred in the context of significant clinical improvement.

A complicating factor in interpreting this study's data is that the SCZ group did not demonstrate specific SF processing impairments, independent of a generalized performance deficit (as revealed in the BSF condition). The only possible exception to this was reduced LSF processing at the discharge assessment, but this effect disappeared when subjects who made a significant number of errors in the BSF condition (where few errors are expected) were removed, suggesting again that some of the initial effect was due to generalized performance impairments (including more variable task engagement). It is also possible that the change in statistical significance level across those 2 analyses was an artifact of lower power after reducing the sample size. Overall, however, the normal SF processing in this study is consistent with the findings of McBain et al.[20] that SF detection is normal, but that this information may get distorted at higher levels of processing (e.g., emotion identification) which were not relevant to the task we used.

How can these results be understood within the context of other prior studies? One possibility is that, as noted by Laprevote and colleagues [17], [18], schizophrenia patients have a bias towards processing LSF stimuli in faces and objects, and, to the extent that this is a feature of the stabilization-stable phases of illness, then as patients move into these phases, face processing would be most disrupted by removal of LSF information. However, this is clearly a post-hoc and speculative hypothesis. What we can say with more certainty is that there is not a generalized LSF processing impairment in schizophrenia, and that more research is needed to clarify under what conditions different spatial frequencies are processed normally or abnormally. Also, independent of spatial frequency type, there was a greater degree of performance decline due to any loss of information for the SCZ group, compared to the other groups, at Time 2, and a trend for this effect to be more pronounced at Time 2 than Time 1. The overall effect is consistent with past findings that image degradation has a stronger effect on schizophrenia patients than other groups [71]–[73], while the possible change over time requires further investigation.

An important finding from this study was that task performance was significantly determined by visual acuity. In particular, poorer visual acuity reduced accuracy on all indices reflecting HSF processing, at both time points, and all of these results survive correction for multiple comparisons. In contrast, there were no relationships between acuity and LSF processing. This is an internally consistent pattern of results since, by definition, HSFs involve finer perceptual distinctions than LSFs. While the groups did not differ in terms of acuity – because we provided corrective lenses to essentially match subjects on optimal acuity – the implications of these findings are that studies that do not match groups on acuity may: 1) confound poor HSF processing with poor acuity; and 2) reveal group differences in HSF processing, or other aspects of perception requiring high resolution, that are artifacts of group differences in acuity. Preliminary evidence consistent with this from this study was that the SCZ-related performance impairment with SF degradation at Time 2 was no longer significant when controlling for visual acuity. While this issue has not received much attention in the cognitive neuroscience of schizophrenia literature, and thus the extent to which many past findings are artifactual is largely unknown, we believe it is critical to attend to, because: 1) impairments in acuity have been noted in children at high risk for schizophrenia [6], [7]; 2) SCZ patients have poorer visual acuity than the general population [28], [29]; 3) SCZ patients as a group are characterized by retinal abnormalities (e.g., retinal ganglion cell axon loss) that can affect acuity [74], [75]; 4) some antipsychotic medications [76], and other medications taken by SCZ patients [76], [77], can blur vision, leading to increased difficulty with HSF [78] processing but leaving LSF processing relatively intact (or enhanced [78]), and, importantly, 5) even small differences in acuity – within the normal range – can affect performance on visual processing tasks on which SCZ patients have performed abnormally in past studies, especially when the elements are composed of higher SFs [79].

A second goal of this study was to compare first-episode and later-episode patients. The performance of both groups was roughly equivalent on SF indices, with the only evidence for a difference being at the discharge time point, where there was a trend for the SCZ group to be more impaired in the BSF-LSF contrast compared to the FEP group. However, on the degradation index, the SCZ group demonstrated significantly more impairment than the FEP group at Time 2. This suggests that the general issue of recognizing degraded images may be something that progresses over time with an increased number of psychotic episodes. However, this hypothesis should be considered cautiously given that the effect was not found at Time 1, it did not remain after controlling for visual acuity, and that some abnormalities in the RT data were observed among FEP subjects (but not hyper-processing of LSF information, as has been found in some studies using traditional grating stimuli [35], [36]). Nevertheless, we believe the progression hypothesis is worth pursuing, since there is other evidence that some perceptual impairments worsen with illness progression [12], [54], as well as cross-sectional evidence from prior studies that LSF processing may shift from hyper- to hypo-active after the first episode in schizophrenia [23], [35], [36], [38], [39].

In a prior study using the same task [24], which also found trend level evidence of poorer LSF processing in schizophrenia, there were significant group differences in fMRI data, indicating excessive fusiform gyrus activity in this group, presumably secondary to poorer quality representations emerging from visual cortex. Therefore, it is also possible that while group differences (e.g., in LSF processing) do exist, the face processing task we used is simply not sensitive enough to detect this in the behavioral data (e.g., lower spatial frequencies may be needed, or there may not be a sufficient number of trials). On the other hand, there was clearly an effect of the SF manipulation in the behavioral data for all groups, and so it is not clear how this could not lead to a differential deficit among SCZ patients if one existed.

Two limitations of the study are noteworthy. First, since we did not include a separate age-matched control group for FEP subjects, it is not clear whether the performance of the FEP group is abnormal compared to age-matched healthy subjects. This is important because low spatial frequency processing can decline with age [80], [81] (although these effects should be minimal within the age range used in this study). Second, the low spatial frequency condition in this study used a level of cycles/degree (2.17) that has been considered medium spatial frequency in some studies [78], although it is half as low as that of medium frequency stimuli used in other studies [14], and close to the LSF value (e.g., 1.5 cycles/degree) used in other studies [66]. Nevertheless, given the general lack of between-group differences in our LSF condition, additional studies using measures involving spatial frequency degraded faces should include lower spatial frequencies.

In conclusion, given the caveats noted above, this study did not find evidence of a specific SF processing impairment in schizophrenia during a face processing task in which SFs were independent of the perceptual discrimination that had to be made (gender discrimination). Moreover, level of SF processing did not change during recovery from an acute phase of illness. There were also no clear differences between first-episode psychosis and later-episode schizophrenia patients in SF processing, although later-episode patients were more affected by overall stimulus degradation at one of the two time points. These data, in the context of prior studies of SF processing in schizophrenia, imply that the conditions under which SF information is processed abnormally are a subset of all possible conditions, and that it remains largely unclear under what circumstances (patient, stimuli, mental state, environmental context, etc.) SF information is processed abnormally relative to psychiatrically healthy subjects. The one clear exception to this statement is that our data are consistent with past findings indicating a lower probability of observing problems in LSF processing when LSF stimuli are presented at suprathreshold levels, as was in the case in this study. On the other hand, the evidence for performance decline with image degradation is consistent with prior studies of degraded stimulus processing in schizophrenia [71], [72]. This suggests that factors other than specific spatial frequencies are involved in the problems that patients have in constructing visual representations of complex stimuli such as faces. We suggest that these factors include poor visual acuity (secondary to schizophrenia and/or medication), as well as intermediate level problems involving structural encoding [82], such as perceptual organization impairments [2], [83], [84].

## Supporting Information

S1 Results **Supplemental results.**
(DOCX)

S1 Data **SPSS Data file.**
(SAV)
