# Supplementary material for: Processing of Spatial-Frequency Altered Faces in Schizophrenia: Effects of Illness Phase and Duration
Source: PLoS One. 2014 Dec 8;9(12):e114642. doi: 10.1371/journal.pone.0114642 (PMC4259337; doi:10.1371/journal.pone.0114642)
Supplement: S1 Results — Supplemental results. (DOCX) [file pone.0114642.s001.docx]

Supplemental Results

Time 1

Accuracy With all subjects included: (ns were FEP=29, SCZ=48, CON=43): LSF *F*(2,119)=1.51, *p*=.23; HSF *F*(2,119)=.62, *p*=.54; sensitivity *F*(2,119)=2.10, *p*=.13; degradation *F*(2,119)=0.01, *p*>.95. With a 61.11% cutoff (ns were FEP=29, SCZ=46, CON=43): LSF *F*(2,117)=1.69, *p*=.19; HSF *F*(2,117)=.39, *p*=.68; sensitivity *F*(2,117)=1.74, *p*=.18; degradation *F*(2,117)=0.47, *p*=.95. With a 90% cutoff (ns were FEP=23, SCZ=30, CON=38): LSF *F*(2,90)=1.41, *p*=.25; HSF *F*(2,90)=0.18, *p*=.84; sensitivity *F*(2,90)=0.89, *p*=.41; degradation *F*(2,90)=0.12, *p*=.89. Finally, there were no differences on any of the accuracy variables (SF, sensitivity, or degradation) between subjects who were present or not present at Time 2 on Time 1 variables: FEP (6 absent vs 23 present) *p*s all > .17; SCZ (12 vs. 36) *p*s all > .32; CON (8 vs. 35) all *p*s > .17.

RT With all subjects included: (ns were same as reported above in accuracy): LSF *F*(2,119)=4.57, *p*<.01; HSF *F*(2,119)=1.36, *p*=.26; sensitivity *F*(2,119)=2.95, *p*=.06; degradation *F*(2,119)=1.78, *p*=.18. With a 61.11% cutoff: LSF *F*(2,117)=4.61, *p*<.02; HSF *F*(2,117)=1.89, *p*=.16; sensitivity *F*(2,117)=3.04, *p*=.05; degradation *F*(2,117)=2.37, *p*<.10. With a 90% cutoff: LSF *F*(2,90)=4.18, *p*<.02; HSF *F*(2,90)=0.82, *p*=.44; sensitivity *F*(2,90)=4.02, *p*<.03; degradation *F*(2,90)=1.03, *p*=.36. Finally, there were no differences on any of the RT variables (SF, sensitivity, or degradation) between subjects who were present or not present at Time 2 on Time 1 variables: FEP (6 absent vs 23 present) *p*s all > .20; SCZ (12 vs. 36) *p*s all > .15; CON (8 vs. 35) all *p*s > .54.

Time 2

Accuracy When the Time 2 data set was restricted to only those subjects scoring above 90% correct in the BSF condition (ns were FEP=18, SCZ=24, CON=34), there were no significant or trend-level effects in either the LSF (*F*(2,77)=1.12, *p*=.33) or HSF (*F*(2,77)=0.75, *p*=.48) conditions, or on the sensitivity index (*F*(2,77)=1.00, p=.37. However, as with the data set restricted to subjects tested at both time points, there was a main effect of group on the degradation index: *F*(2, 77)=6.82, *p*=.002*.* Post-hoc Scheffé tests again indicated that at Time 2, the SCZ group demonstrated more overall performance impairment when SF information was removed from the facial images, compared to the FEP (p<.02) and CON (p<.01) groups, who did not differ from each other (*p*=.96).

Correlations between symptoms and task performance

Symptom correlates (Spearman *rho*) of performance were examined, using the BSF-HSF, and BSF-LSF difference scores. For the patient group as a whole, at Time 1, no values reached significance (all *r*_s_ values <.21 (absolute values); *p*s > .14). Values were similar at Time 2 (all *r*_s_ values <.22; *p*s > .11). For the FEP group alone, at Time 1, there were no significant correlations (*r*_s_ values <.34; *p*s > .13). At Time 2, there were significant positive correlations between PANSS Excitement symptom scores and lack of performance decrement in the LSF condition *r*_s_=.57, *p*=.009, and between scores on Cuesta and Peralta’s 3-item Disorganization factor[^55^](#_ENREF_55) and LSF scores *r*_s_=.56; *p*=.01. All other *r*_s_ values were > .32, and p values were > .17. For the SCZ group, at Time 1, there were no significant correlations (all *r*_s_ values <.31; *p*s > .09). The same occurred at Time 2 (all *r*_s_ values <.28; *p*s > .10).

The sensitivity and degradation indices did not correlate significantly with any symptom factor for either group alone at Time 1 (all *r*_s_ values <.32; *p*s > .06). For the patient group combined, the only significant correlation was between PANSS Depression factor scores and the degradation index: *r*_s_=.28; *p*=.04. All other *r*_s_ values <.24; *p*s > .07. At Time 2, for the SCZ group, the sole significant correlation was between PANSS Excitement factor scores and the sensitivity index: *r*_s_=.34; *p*=.04. All other *r*_s_ values <.29; *p*s > .09. For the FEP group, there was a significant negative correlation between PANSS Depression scores and sensitivity index scores: *r*_s_=-.50; *p*=.02, indicating that higher depression levels were associated with less sensitivity to information removal from images. All other *r*_s_ values <.42; *p*s > .06. For the patient group combined, no correlations were significant (all *r*_s_ values <.17; *p*s > .20).

Correlations between medication dosages and task performance

At Time 1, for the FEP group, CPZ equivalent dose was related to sensitivity (*r*_s_=-.42, *p*<.04) but not degradation (*r*_s_=.22, *p*=.29) index scores. The former result indicates that FEP patients who showed the largest difference between LSF and HSF condition performance were on lower doses of medication compared to FEP patients whose scores in these conditions were more similar. Since this was the only significant correlation out of all correlations performed between task scores and medication data (see below), and since it would not survive even the most liberal correction for multiple comparisons, it should be considered with caution.

Because only 5 FEP patients were taking benzodiazepines, no correlations were calculated between task performance and diazepam equivalent dosage. For the SCZ group, CPZ equivalent dose was unrelated to sensitivity (*r*_s_=-.24, p=.10) and degradation (*r*_s_=.08, p=.59) index scores. Because only 6 SCZ patients were taking benzodiazepines, no correlations were calculated between task performance and diazepam equivalent dosage.

At Time 1, for the patient group as a whole, CPZ equivalent dose was unrelated to sensitivity (*r*_s_=-.22, *p*=.06) and degradation (*r*_s_=.10, *p*=.39) index scores. Diazepam equivalent dose was also unrelated to sensitivity (*r*_s_=.17, *p*=.62) and degradation (*r*_s_=.36, *p*=.28) index scores. Note that only 11 patients were taking benzodiazepines, and so the latter analyses are based on a very small sample.

At Time 2, for the FEP group, CPZ equivalent dose was unrelated to sensitivity (*r*_s_=.04, *p*=.86) and degradation (*r*_s_=-.29, p=.19) index scores. Because only 4 FEP patients were taking benzodiazepines, no correlations were calculated between task performance and diazepam equivalent dosage. For the SCZ group, CPZ equivalent dose was unrelated to sensitivity (*r*_s_=.04, *p*=.86) and degradation (*r*_s_=-.29, *p*=.19) index scores. Because only 4 SCZ patients were taking benzodiazepines, no correlations were calculated between task performance and diazepam equivalent dosage.

At Time 2, for the patient group as a whole, CPZ equivalent dose was unrelated to sensitivity (*r*_s_=-.07, *p*=.56) and degradation (*r*_s_=.13, *p*=.34) index scores. Diazepam equivalent dose was also unrelated to sensitivity (*r*_s_=-.24, *p*=.51) and degradation (*r*_s_=-.15, *p*=.68) index scores. Note that only 10 patients were taking benzodiazepines, and so the latter analyses are based on a very small sample.

For the patient sample as a whole, mean CPZ equivalent dosage increased from Time 1 (289 mg, SD=207) to Time 2 (391 mg, SD=377): *t*(63)=-2.43, *p*<.02. Mean diazepam equivalent dosage remained identical across both time points (7.95 at both points; SD at Time 1=6.31, SD at Time 2=5.46). Below, correlations between change in sensitivity and degradation accuracy index scores over time, and change in CPZ equivalent dose over time, are presented. Because diazepam equivalent dosages did not change over time, correlations with degrees of change in task indices were not calculated for this variable.

For the FEP group, there were no relationships between change in CPZ equivalent dose from Time 1 to Time 2, and change in sensitivity (*r*_s_=.27, *p*=.24) or degradation (*r*_s_=.19, *p*=.32) index values. Correlations were also non-significant for the SCZ group (sensitivity *r*_s_=-.19, *p*=.27; degradation *r*_s_=.15, *p*=.39). For both patient groups combined, there were no relationships between change in CPZ equivalent dosage from Time 1 to Time 2, and change in sensitivity (*r*_s_=-.04, *p*=.78) or degradation (*r*_s_=-.19, p=.16) index values.
